# Dentistry Not a Specialty of Medicine

**Published:** 1887-01

**Authors:** Norman W. Kingsley

**Affiliations:** President of the New York State Dental Society


### Selections.

### Dentistry not a Specialty of Medicine.

Following this will be found two letters, one from the *Medical Record* of November 20th, the other from the same journal of December 4th, in which both sides of a much discussed question are considered, the thoughts presented in each are well worthy of attention.

The question is one in certain aspects, involving considerable importance, it cannot, however, be settled by the mere *ipse dixit* of a few persons, but a decision will be wrought out, in that which is done, rather than by what is said.—[Ed. Register.]

To The Editor of The Medical Record.

*Sir:* —The leading article of the Medical Record for October 23d, by W. A. Purrington, Esq., upon the statutes relating to the practice of medicine, incites me to correct a somewhat prevalent but erroneous impression, viz., that dentistry is a specialty of medicine.

That there may be no ambiguity, let me state that by the term dentistry I mean to cover every branch and department known under that name, and a dentist in the full sense of the term in the present stage of the art is one who understands and can practice each and every specialty of it.

A dentist may be an oral surgeon, but an oral surgeon is not a dentist. A dentist may be an excellent anatomist, physiologist, chemist, microscopist, artist, or mechanic, but no one of these practiced to perfection makes him a dentist.

Oral surgery, as practiced by dentists, is only a very small, but not unimportant, specialty of dentistry; it occupies the debatable ground between dental and general surgery, but as an essential department of practical dentistry its importance has been magnified by those practitioners who are more skilled in surgery than they are in dentistry.

Oral surgery, even in its most comprehensive sense, is not dentistry, any more than dentistry in its most comprehensive sense is only oral surgery.

I therefore affirm that dentistry *is not* a specialty of any other science or art, but is a profession in itself, as separate and distinct from all others as any other calling or vocation is distinct from any other.

Dentistry is a profession, because it is a vocation of beneficence. This is so patent that I need not attempt to prove it or enlarge upon it. Millions are on the earth to-day who call us blessed, because of the comfort we have given them and the benefit they have derived from us.

Dentistry is a profession by universal acknowledgment; it has been an organized science for more than a generation, and has been called a "profession" by common consent by the cultured and uncultured as well as by its own practitioners.

Even the highest authorities in medical literature refer to dentistry, not as the "dental specialty of medicine," but as a "profession."

The designation of it as a "profession" is not an assumption like that of the barber, the dancing-master, or the itinerant phrenologist; it is entitled to the distinction because the mastery of it as a science or an art involves a considerable knowledge of many other sciences.

Its resources are not only nearly all the sciences, but in an equal degree nearly all the arts. Hardly an art, from plumbing to sculpture, but has its prototype in some branch of dentistry, and yet it is not a department or specialty of any one of them.

While a large part of its processes are of a mechanical nature, it is not a mechanical trade, inasmuch as a mechanical trade is governed by fixed rules and a routine of labor, in which each workman is a servile imitator of the pattern given him, and can become master of his trade without any knowledge beyond its details.

His brain is not constantly called upon to apply established principles to entirely new conditions and surrounding circumstances—the distinction which I would make between a trade and a profession being, that while the latter may employ the identical methods of the former, the judgment and the inventive faculties of the practitioner must be in active exercise to apply those principles and those methods to constantly varying conditions. The predominating feature and characteristic of dentistry, that which removes it farther than all else combined from medicine, is the mechanical nature of its methods. So much alike are the methods of the gold and silver jeweler to dentistry, that the acquirement of one would be considerable of an education for the other. Yet making gold and silver jewelery is not a profession; it is a trade. Dentistry, while using the same mechanical processes, is obliged to add invention to their application to every case. The *methods* of the painter and the sculptor are the methods of the mechanic. But portrait, figure, and landscape painting and sculpture are branches of fine art, and the vocation is a *profession*, not a trade. That which dignifies the practice of dentistry, bringing it above ordinary mechanics, is the fact that the operations are performed upon living organisms, and that which makes it professional is the knowledge of anatomy, pathology, etc., which discriminates in directing the mechanical treatment. Dentistry is not a specialty of medicine, because its chief and predominating characteristics are utterly unlike anything which is taught in medicine, requiring for their successful performance natural faculties and acquirements that are entirely distinct from the practice of medicine. That which makes dentistry as a science kindred to medicine as a science, is the fact that it deals with a small but important part of the human economy. But the equally great fact that its methods are entirely distinct, requiring special education and special training, makes it an independent science and in no sense subordinate to the other. The training of a dental student for his professional career is totally unlike that required by a medical student. Medicine involves hospital and bedside practice, but dentistry involves, along with the study of the sciences, training of the fingers, first, second, and all the time.

Dentistry became an independent professian, not through any spirit of rebellion against the medical profession, but from sheer necessity. The fathers of dentistry in this country were graduates of medicine, and hoped to dignify their vocation by grafting it upon medicine and have the theory and practice taught in medical schools. Their application was refused, and the history of dentistry as an independent progressive and scientific organization began, and to-day the wondrous fact is the astonishment and admiration of the scientific world. We have more than a dozen independent institutions of learning which teach everything that a dentist need know, and in separate institutions, and no other, can a dental student obtain that knowledge. Those universities which teach dentistry teach it as a separate department from medicine, and confer a distinct degree. We have an independent literature, which is not indebted to medicine so much as it is to other sciences. Anatomy, physiology, histology, microscopy, chemistry, etc., are not medical studies. They are sciences, upon which medical and other studies are based.

We have an independent journalism larger than the total of medical journalism when our history began. We have independent national, state, and local organizations that are vital, active, and progressive; and what might once have been by dentistry taught and practised as a specialty of medicine cannot now in the very nature of things ever be brought about. If to-day all the medical colleges, together with the entire medical profession, were blotted out, the practice of dentistry would not be injured in the least; nor would humanity suffering from diseases of the teeth be one whit the less cared for. Dentistry has come to stay—not as a specialty, but as an honorable, dignified, learned, scientific, beneficent, and independent profession. Dental colleges have come to stay, and the degree of D.D.S. has come to stay. * * * *

NORMAN W. KINGSLEY, D.D.S.
*President of the New York State Dental Society.*

---

